# The microRNA signature of patients with sunitinib failure: regulation of *UHRF1* pathways by *microRNA-101* in renal cell carcinoma

**DOI:** 10.18632/oncotarget.10887

**Published:** 2016-07-28

**Authors:** Yusuke Goto, Akira Kurozumi, Nijiro Nohata, Satoko Kojima, Ryosuke Matsushita, Hirofumi Yoshino, Kazuto Yamazaki, Yasuo Ishida, Tomohiko Ichikawa, Yukio Naya, Naohiko Seki

**Affiliations:** ^1^ Department of Functional Genomics, Chiba University Graduate School of Medicine, Chiba, Japan; ^2^ Department of Urology, Chiba University Graduate School of Medicine, Chiba, Japan; ^3^ Moores Cancer Center, University of California, San Diego, La Jolla, CA, United States of America; ^4^ Department of Urology, Teikyo University Chiba Medical Center, Chiba, Japan; ^5^ Department of Urology, Graduate School of Medical and Dental Sciences, Kagoshima University, Kagoshima, Japan; ^6^ Department of Pathology, Teikyo University Chiba Medical Center, Chiba, Japan

**Keywords:** microRNA, renal cell carcinoma, sunitinib, miR-101, UHRF1

## Abstract

Molecular targeted therapy is a standard treatment for patients with advanced renal cell carcinoma (RCC). Sunitinib is one of the most common molecular-targeted drugs for metastatic RCC. Molecular mechanisms of sunitinib resistance in RCC cells is still ambiguous. The microRNA (miRNA) expression signature of patients with sunitinib failure in RCC was constructed using a polymerase chain reaction (PCR)-based array. Several miRNAs that were aberrantly expressed in RCC tissues from patients treated with sunitinib were identified in this analysis. *MicroRNA-101 (miR- 101)* was markedly suppressed in sunitinib treated RCC tissues. Restoration of *miR-101* significantly inhibited cell migration and invasion in Caki-1 and 786-O cells. Ubiquitin-like with PHD and ring finger domains 1 (*UHRF1*) was directly suppressed by *miR-101* in RCC cells, and overexpression of UHRF1 was confirmed in sunitinib-treated RCC tissues. The pathways of nucleotide excision repair and mismatch repair were significantly suppressed by knockdown of *UHRF1*. Our findings showed that antitumor *miR-101*- mediated *UHRF1* pathways may be suppressed by sunitinib treatment.

## INTRODUCTION

Renal cell carcinoma (RCC) accounts for over 80% of kidney cancers, and 338,000 new cases were diagnosed worldwide in 2012 [1]. The incidence of RCC is increasing due to recent improvements in screening technologies, such as ultrasound and computed tomography. Although molecular-targeted anti-angiogenic multi-tyrosine kinase inhibitors have been developed, they show limited effects, particularly in patients with advanced RCC; consequently, the prognosis of advanced-stage RCC is still poor [2].

Sunitinib is one of the most common molecular-targeted drugs for metastatic RCC. A phase 3 clinical trial of sunitinib versus interferon alpha in patients with metastatic RCC ushered in the molecular-targeted era in the treatment of RCC [3]. Although side effects, such as hand-foot syndrome, thrombocytopenia, general fatigue, and hypothyroidism, often occur with sunitinib treatment, sunitinib is still a standard treatment for metastatic RCC due to the relatively longer progression-free survival time and higher response rate [4–6]. Additionally, sunitinib therapy is often associated with treatment failure in patients with metastatic RCC.

MicroRNAs (miRNAs) are small noncoding RNAs that function as a fine tuner of protein-coding or noncoding gene expression [7, 8]. A growing body of evidence suggests that aberrantly expressed miRNAs contribute to cancer pathogenesis and drug resistance [9, 10]. We have sequentially identified antitumor miRNA-mediated RCC pathways based on RCC miRNA signatures [11–13]. The next challenge in our RCC study is to identify key molecules and novel pathways involved in the resistance of molecular-targeted therapies for RCC.

Accordingly, in this study, we constructed a miRNA expression signature to identify pathways activated by sunitinib treatment using autopsy specimens from patients with RCC. The miRNA expression signature revealed that *microRNA-101*(*miR-101*) was significantly suppressed in sunitinib-treated RCC tissues compared with that in primary RCC tissues. Additionally, we demonstrated that *miR-101* exhibited antitumor activity and directly suppressed ubiquitin-like with PHD and ring finger domains 1 (*UHRF1*). Moreover, we investigated *UHRF1*-mediated downstream pathways in RCC cells. Elucidation of the miRNA signature of sunitinib-treated RCC tissues may be useful for identification of the novel molecular mechanisms of RCC recurrence, metastasis, and drug resistance.

## RESULTS

### Construction of the miRNA expression signature of sunitinib-treated RCC

First, we analyzed the expression levels of mature miRNAs in sunitinib-treated RCC specimens by PCR- based array analysis. Also, we reviewed our miRNA expression data of 10 sunitinib-naïve RCC specimens and 5 normal kidney tissues, and we constructed a signature of downregulated miRNAs in sunitinib-naïve RCC tissues (Supplementary Table S1). Of the 11 sunitinib- treated RCC specimens examined in this study, we used 4 specimens (No. 1, No. 5, No. 6, and No. 8; Table 1) for array analysis. Compared with our previous miRNA signature in primary RCC specimens, 232 miRNAs were significantly downregulated in sunitinib-treated RCC specimens [11]. We listed the top 40 downregulated miRNAs in sunitinib-treated RCC specimens (Table 2). Among them, we focused on *miR-101*, which showed the most dramatic downregulation in sunitinib-treated RCC, for further studies.

**Table 1:** Patient characteristics (sunitinib-treated RCC specimens)

Patient	Specimen No.	Location	Age (years)	stage at diagnosis				Histological type	Grade	Treatment	Treatment duration (months)	Pathological feature of autopsy	survival from diagnosis (months)
				Stage	cT	cN	cM						
A	1	Kidney	69	4	4	2	1	Clear cell carcinoma	3	Sunitinib, temsirolimus	8.5	Multiple lung metastasis Bone metastasis	9.1
	2	Lymph node											
	3	Liver											
	4	Lung											
	5	Tumor emboli											
B	6	Kidney	80	3	3c	0	0	Clear cell carcinoma	3	Sunitinib	0.7	IVC tumor emboli	1.8
	7	Kidney											
	8	Tumor emboli											
C	9	Mesenterium	62	1	1b	0	0	Clear cell carcinomawith spindle cell carcinoma	3	Sunitinib, axitinib	34	Multible bone metastasis Pleural metastasis Lung metastasis Paraaorta lymph node metastasis	43
	10	Lymph node											
	11	Pleura											

**Table 2:** Downregulated miRNAs in sunitinib-treated RCC (versus primary RCC)

miRNA	Log2 ratio (sunitinib failure/primary)	Primary RCC	Sunitinib failure RCC	*P*-value
*hsa-miR-101*	–9.89	0.00193	2.04E-06	6.74E-05
*hsa-miR-29b*	–9.73	0.00213	2.51E-06	6.20E-05
*hsa-miR-190*	–9.66	0.00061	7.51E-07	6.39E-04
*hsa-miR-128*	–9.46	0.00036	5.11E-07	2.52E-04
*hsa-miR-23b*	–9.40	0.00035	5.11E-07	2.80E-03
*hsa-miR-10b*	–9.36	0.00599	9.10E-06	3.84E-04
*hsa-miR-766*	–9.27	0.00032	5.11E-07	1.75E-03
*hsa-miR-142-5p*	–9.22	0.00035	5.92E-07	9.89E-04
*hsa-miR-1275*	–9.03	0.00027	5.11E-07	2.17E-03
*hsa-miR-629*	–8.99	0.00026	5.11E-07	3.24E-04
*hsa-let-7c*	–8.91	0.00216	4.49E-06	3.73E-04
*hsa-miR-135a*	–8.89	0.0006	1.26E-06	3.77E-02
*hsa-miR-320b*	–8.84	0.00083	1.81E-06	7.83E-05
hsa-miR-24-2*	–8.82	0.00023	5.11E-07	2.68E-04
hsa-miR-99a*	–8.67	0.00035	8.60E-07	2.96E-04
hsa-miR-31*	–8.59	0.00112	2.92E-06	4.68E-02
*hsa-miR-450a*	–8.57	0.00019	5.11E-07	1.33E-03
*hsa-miR-455-3p*	–8.49	0.00173	4.80E-06	5.45E-05
*hsa-miR-340*	–8.43	0.00448	1.30E-05	6.99E-08
hsa-miR-126*	–8.37	0.09059	2.75E-04	1.85E-04
hsa-miR-15b*	–8.35	0.00017	5.11E-07	8.21E-03
*hsa-miR-29c*	–8.32	0.03819	1.20E-04	1.13E-04
*hsa-miR-204*	–8.29	0.03376	1.08E-04	6.10E-03
hsa-miR-424*	–8.14	0.00014	5.11E-07	2.59E-03
*hsa-miR-27b*	–8.13	0.00574	2.05E-05	2.67E-04
*hsa-miR-1*	–8.08	0.00014	5.11E-07	5.84E-03
*hsa-miR-592*	–8.05	0.00111	4.19E-06	2.77E-03
hsa-miR-20a*	–7.97	0.0003	1.21E-06	4.21E-04
*hsa-miR-450b-5p*	–7.85	0.00024	1.02E-06	1.48E-05
*hsa-miR-10a*	–7.69	0.00458	2.22E-05	1.95E-04
*hsa-let-7a*	–7.64	0.00517	2.60E-05	3.20E-06
*hsa-miR-32*	–7.63	0.00014	7.23E-07	1.13E-04
*hsa-miR-324-5p*	–7.63	0.00079	3.98E-06	1.07E-04
*hsa-miR-429*	–7.63	0.0042	2.12E-05	5.17E-04
*hsa-miR-98*	–7.61	0.00055	2.80E-06	1.39E-06
*hsa-miR-598*	–7.59	0.00105	5.42E-06	5.39E-06
*hsa-miR-148b*	–7.57	0.00042	2.20E-06	8.74E-07
*hsa-miR-577*	–7.56	0.00021	1.11E-06	7.18E-03
*hsa-miR-26b*	–7.51	0.05631	3.10E-04	3.85E-06
*hsa-miR-545*	–7.48	0.00018	1.03E-06	3.89E-05

### Expression levels of miR-101 in RCC clinical specimens and RCC cell lines

Using RT-qPCR, we evaluated the expression levels of *miR-101* in normal kidney (*n* = 41), primary RCC (*n* = 42), and sunitinib-treated RCC (*n* = 11) tissues. Normal kidney tissues were adjacent to primary RCC tissues. The histological type of all primary RCC specimens was clear cell RCC, and 81.0% of patient tissues were classified as pT1 tumors according to the TNM classification (Table 3).

**Table 3:** Patient characteristics (primary RCC specimens)

Total number	42	
Median age (range) (years)	69	(41–91)
Sex		
Male	30	71%
Female	12	29%
Laterality		
Right	20	48%
Left	21	50%
Bilateral	1	2%
Histology		
Clear cell RCC	42	100%
Tumor grade		
G1	5	12%
G2	29	69%
G3	7	2%
Unknown	1	2%
Pathological tumor stage		
pT1	34	81%
pT2	1	2%
pT3	6	14%
Unknown	1	2%
Metastasis		
M 0	37	88%
M 1	5	12%
Venous invasion		
v 0	26	62%
v 1	15	36%
Unknown	1	2%
Recurrence		
Recurrence +	3	7%
Recurrence –	26	62%
Unknown	13	31%

**Figure 1:**
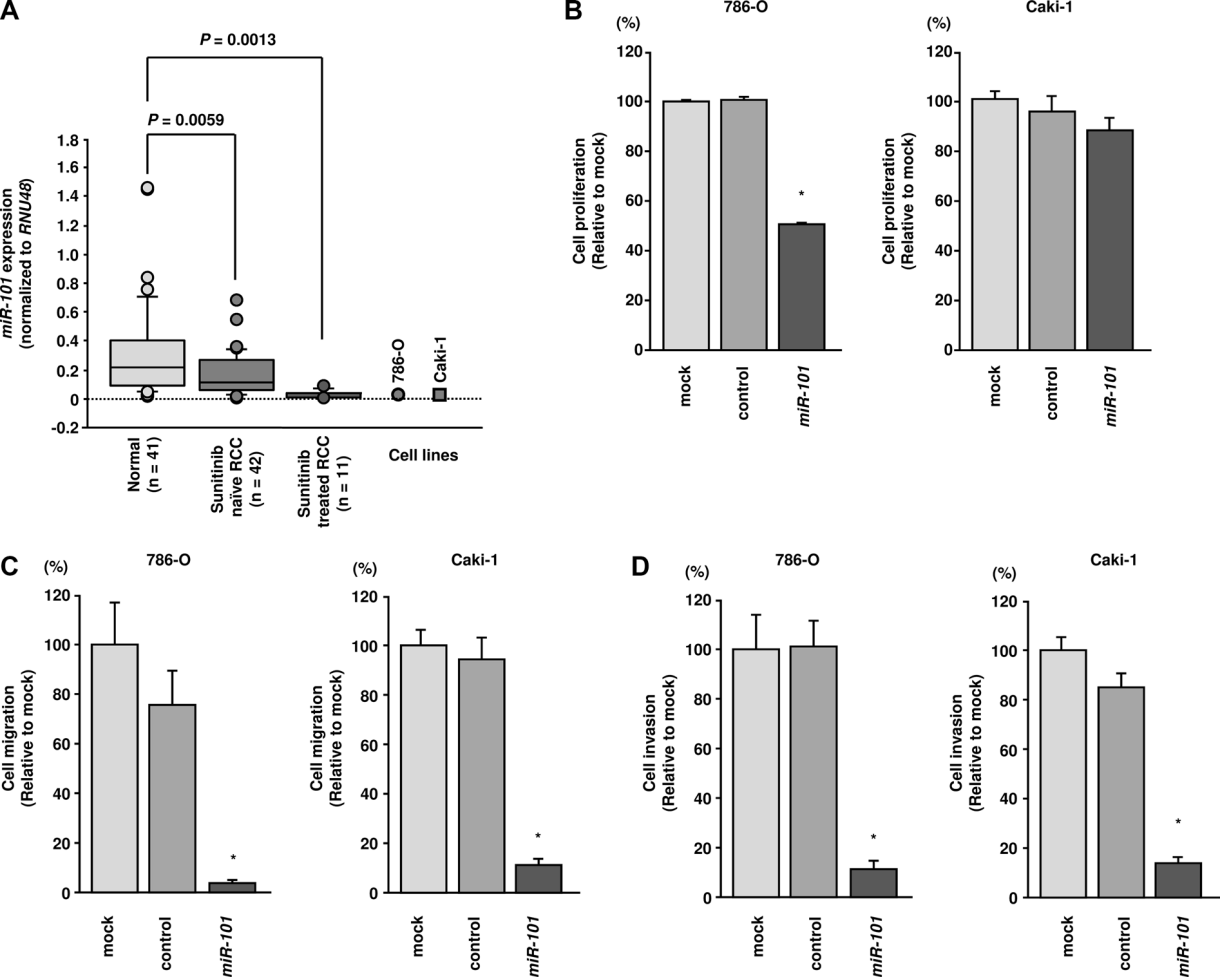
Analysis of *miR-101* expression in RCC clinical specimens and functional analysis of *miR-101* transfection in 786-O and Caki-1 cells (**A**) Expression levels of *miR-101* in RCC clinical specimens. *RNU48* was used for normalization. (**B**) Cell proliferation was assessed 72 h after transfection with *miR-101* using XTT assays. (**C**) Cell migration was assessed 48 h after transfection with *miR-101* using uncoated Transwell polycarbonate membrane filters. (**D**) Cell invasion was assessed 48 h after transfection with *miR- 101* using Matrigel invasion assays. *P< 0.0001. The bars indicate SDs.

The expression levels of *miR-101* were significantly downregulated (*P* = 0.022) in primary RCC tissues compared with that in normal kidney tissues (Figure 1A). Furthermore, the levels of *miR-101* were significantly downregulated (*P* = 0.0013) in sunitinib-treated RCC tissues compared with those in normal kidney tissues (Figure 1A). *miR-101* expression levels were low in the RCC cell lines 786-O and Caki-1.

### Effects of restoring miR-101 expression on cell proliferation, migration, and invasion in RCC cells

To investigate the functional roles of *miR-101* in RCC, we performed gain-of-function studies in 786-O and Caki-1 cells by transfecting the cells with miRNA mimics.

XTT assays indicated that cell proliferation was inhibited by *miR-101* transfection in 786-O cells but not in Caki-1 cells (*P* < 0.0001; Figure 1B). Using wound-healing assays, *miR-101* transfection significantly inhibited cell migration as compared with mock- or miR-control-transfected cells (*P* < 0.0001; Figure 1C). Similarly, Matrigel invasion assays demonstrated that cell invasion activity was significantly inhibited in *miR-101* transfectants in comparison with mock or miR-control transfectants (*P* < 0.0001; Figure 1D).

### Identification of target genes suppressed by miR- 101 in RCC

To identify target genes of *miR-101*, we performed *in silico* analysis with the TargetScan program and GEO database. Analysis by the TargetScan program demonstrated that *miR-101* could target 3,013 genes according to the sequences of their 3′UTRs. Among these genes, 790 had broadly conserved *miR-101* sites across vertebrates. To gain further insights into which genes were suppressed by tumour-suppressive *miR-101* in RCC, we investigated their expression statuses in RCC clinical specimens and examined gene expression profiles in the GEO database (accession numbers: GSE36985 and GSE22541) to evaluate upregulated genes in RCC specimens. Consequently, among the 790 putative conserved target genes of *miR-101*, 43 genes were significantly upregulated in RCC specimens compared with those in normal kidney tissues (log_2_ ratio > 1.0). We sorted these candidate genes in order of expression levels in RCC from the GEO database, because genes with high expression in RCC tissues are thought to function as oncogenes in RCC. Among genes which have conserved target sites for *miR-101*, *UHRF1* was the most upregulated gene. Among genes with multiple conserved target sites for *miR-101, EZH2* showed the greatest upregulation (Table 4).

**Table 4:** Putative target genes of *miR-101* and upregulated genes in RCC clinical specimens

Entrez gene ID	Symbol	Location	Gene name	No. of conserved sites	No. of poorly conserved sites	GEO fold change
29128	*UHRF1*	19p13.3	ubiquitin-like with PHD and ring finger domains 1	1	0	3.178567
8497	*PPFIA4*	1q32.1	protein tyrosine phosphatase, receptor type, f polypeptide (PTPRF), interacting protein (liprin), alpha 4	1	0	3.09998
1404	*HAPLN1*	5q14.3	hyaluronan and proteoglycan link protein 1	1	0	2.781324
6664	*SOX11*	2p25.2	SRY (sex determining region Y)-box 11	1	0	2.577679
163404	*LPPR5*	1p21.3	lipid phosphate phosphatase-related protein type 5	1	0	2.450066
2335	*FN1*	2q35	fibronectin 1	1	1	2.446963
23023	*TMCC1*	3q22.1	transmembrane and coiled-coil domain family 1	1	0	2.226072
286336	*FAM78A*	9q34.13	family with sequence similarity 78, member A	1	1	2.194299
2146	*EZH2*	7q36.1	enhancer of zeste homolog 2 (Drosophila)	2	0	2.003227
5129	*CDK18*	1q32.1	cyclin-dependent kinase 18	1	0	2.002138
54541	*DDIT4*	10q22.1	DNA-damage-inducible transcript 4	1	0	1.998703
55824	*PAG1*	8q21.13	phosphoprotein associated with glycosphingolipid microdomains 1	1	0	1.995799
114088	*TRIM9*	14q22.1	tripartite motif containing 9	1	0	1.912238
23452	*ANGPTL2*	9q33.3	angiopoietin-like 2	1	0	1.707627
3782	*KCNN3*	1q21.3	potassium intermediate/small conductance calcium-activated channel, subfamily N, member 3	2	1	1.643804
10019	*SH2B3*	12q24.12	SH2B adaptor protein 3	1	0	1.578665
54329	*GPR85*	7q31.1	G protein-coupled receptor 85	1	0	1.54526
84206	*MEX3B*	15q25.2	mex-3 homolog B (C. elegans)	1	0	1.526703
50515	*CHST11*	12q23.3	carbohydrate (chondroitin 4) sulfotransferase 11	1	1	1.521547
2697	*GJA1*	6q22.31	gap junction protein, alpha 1, 43kDa	1	0	1.484238
6925	*TCF4*	18q21.2	transcription factor 4	2	0	1.474271
60675	*PROK2*	3p13	prokineticin 2	1	0	1.455421
23551	*RASD2*	22q12.3	RASD family, member 2	1	0	1.436602
23151	*GRAMD4*	22q13.31	GRAM domain containing 4	1	0	1.424502
1003	*CDH5*	16q21	cadherin 5, type 2 (vascular endothelium)	1	0	1.387176
4233	*MET*	7q31.2	met proto-oncogene (hepatocyte growth factor receptor)	1	0	1.35197
2313	*FLI1*	11q24.3	Friend leukemia virus integration 1	1	0	1.324487
7039	*TGFA*	2p13.3	transforming growth factor, alpha	1	0	1.320208
2113	*ETS1*	11q24.3	v-ets erythroblastosis virus E26 oncogene homolog 1 (avian)	1	0	1.319352
64919	*BCL11B*	14q32.2	B-cell CLL/lymphoma 11B (zinc finger protein)	1	0	1.312486
491	*ATP2B2*	3p25.3	ATPase, Ca++ transporting, plasma membrane 2	2	0	1.306825
3832	*KIF11*	10q23.33	kinesin family member 11	1	0	1.299276
114800	*CCDC85A*	2p16.1	coiled-coil domain containing 85A	1	0	1.22271
111	*ADCY5*	3q21.1	adenylate cyclase 5	1	0	1.214093
80149	*ZC3H12A*	1p34.3	zinc finger CCCH-type containing 12A	1	0	1.203416
50807	*ASAP1*	8q24.21	ArfGAP with SH3 domain, ankyrin repeat and PH domain 1	1	1	1.145483
2200	*FBN1*	15q21.1	fibrillin 1	1	0	1.11977
54877	*ZCCHC2*	18q21.33	zinc finger, CCHC domain containing 2	1	0	1.118277
861	*RUNX1*	21q22.12	runt-related transcription factor 1	2	0	1.105534
84627	*ZNF469*	16q24.2	zinc finger protein 469	1	0	1.100592
80727	*TTYH3*	7p22.3	tweety homolog 3 (Drosophila)	1	0	1.096306
23295	*MGRN1*	16p13.3	mahogunin, ring finger 1	1	0	1.030189
162073	*ITPRIPL2*	16p12.3	inositol 1,4,5-trisphosphate receptor interacting protein-like 2	1	1	1.024236

**Figure 2:**
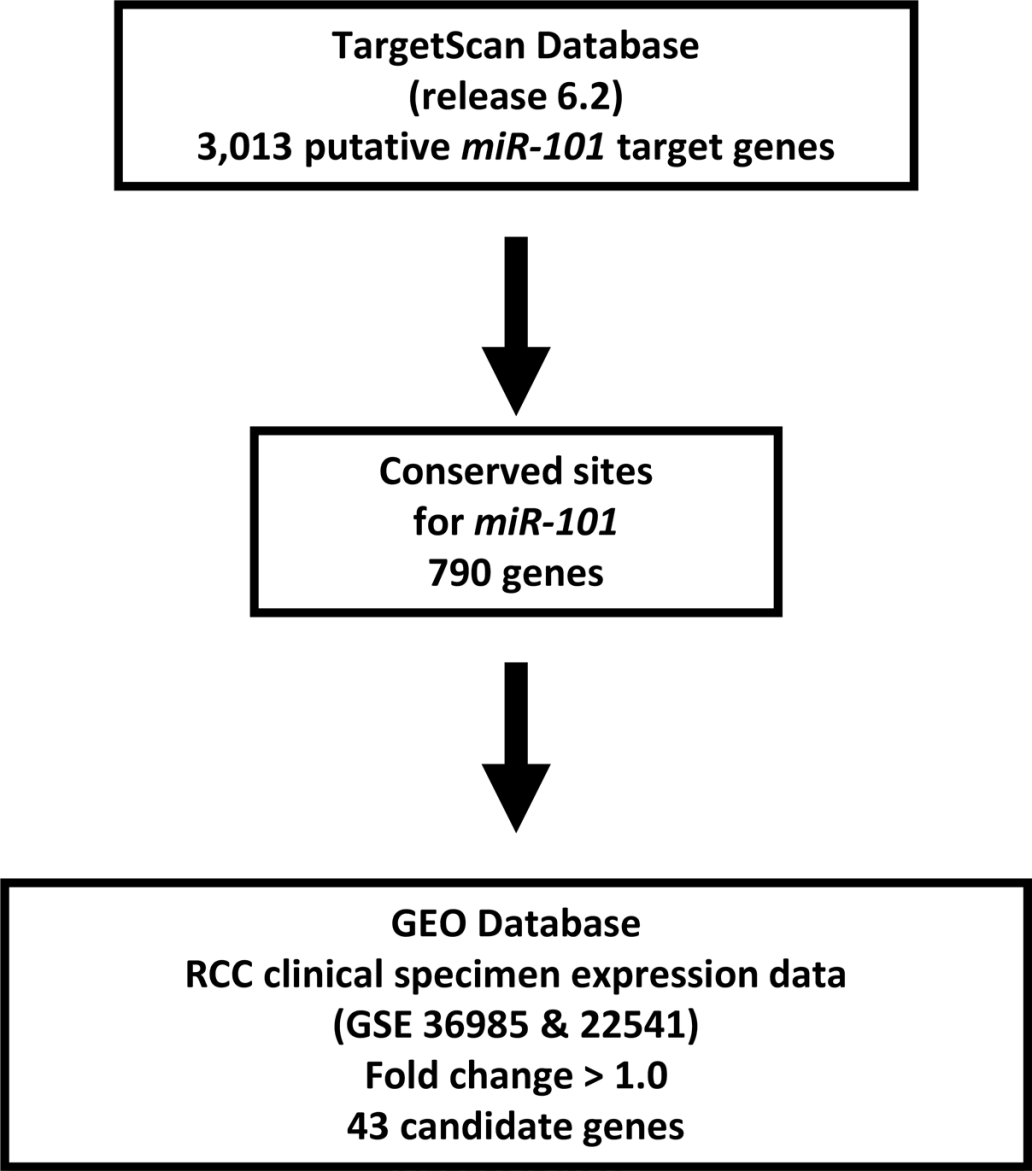
Selection strategy for target genes of *miR-101.* Analysis using the TargetScan program showed that 3,013 genes had putative target sites for *miR-101* in their 3′ UTRs. Among these genes, 790 had conserved target sites among vertebrates for *miR-101*. Then, we assessed the expression levels of these genes in RCC clinical specimens using GEO expression profiles (GSE36985 and GSE22541). Finally, genes upregulated in RCC (log_2_ ratio > 1.0) were selected as putative target genes.

Thus, we focused on *UHRF1* and *EZH2* for further studies. Our strategy for selection of *miR-101*-targeted genes is shown in Figure 2.

### UHRF1 and EZH2 were downregulated by miR- 101 transfection in RCC cells

**Figure 3:**
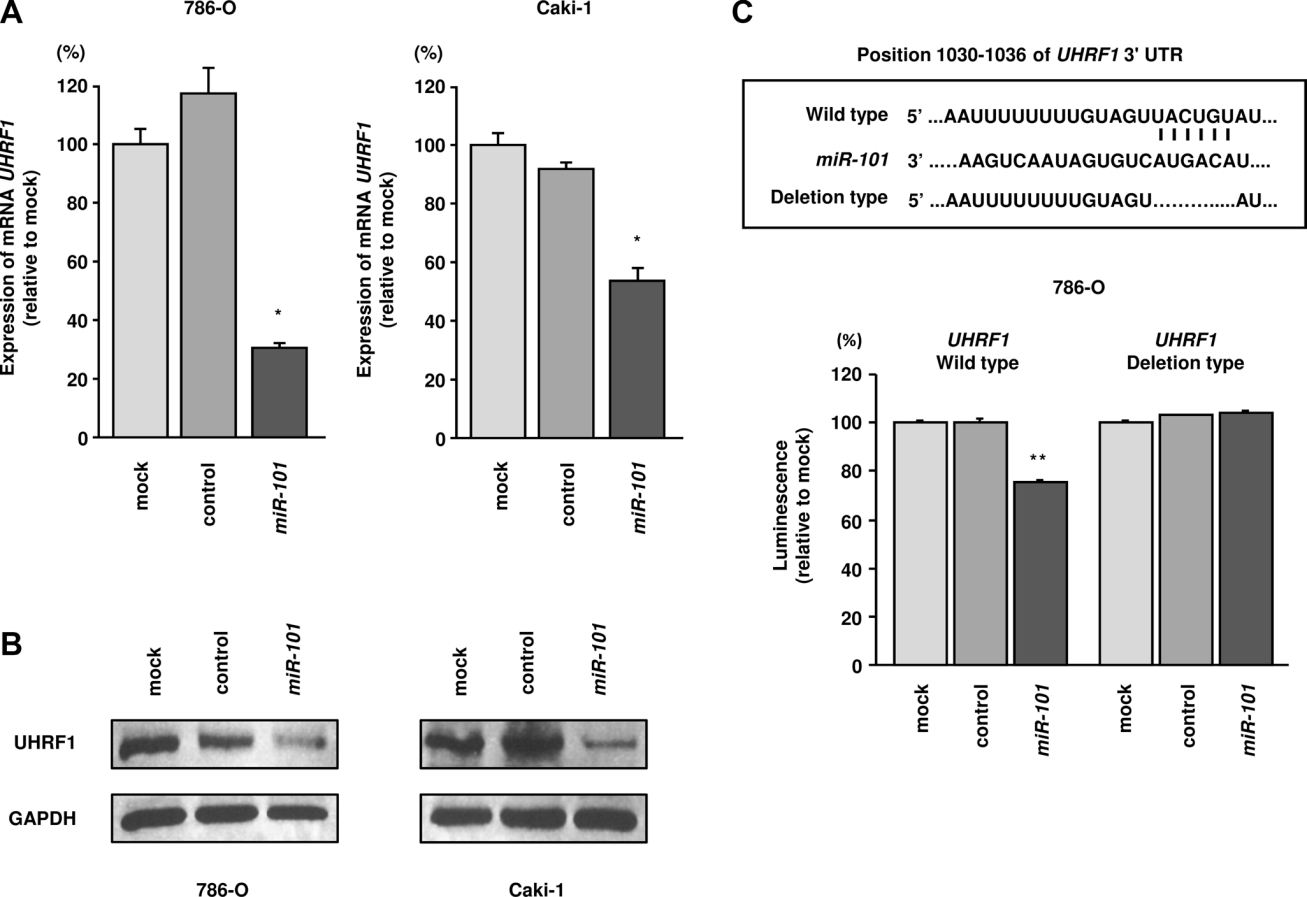
*miR-101* directly downregulated UHRF1 expression in RCC cells (**A**) *UHRF1* mRNA expression 72 h after transfection with *miR-101*. *GUSB* was used as an internal control. (**B**) UHRF1 protein expression 72 h after transfection with *miR-101*. GAPDH was used as a loading control. (**C**) *miR-101* binding sites in *UHRF1* mRNA. Luciferase reporter assays were carried out using a vector encoding the putative *miR-101* target site in the *UHRF1* 3′-UTR (position 1030–1036) for wild-type and deletion constructs. *P < 0.002, **P < 0.0001. The bars indicate SDs.

**Figure 4:**
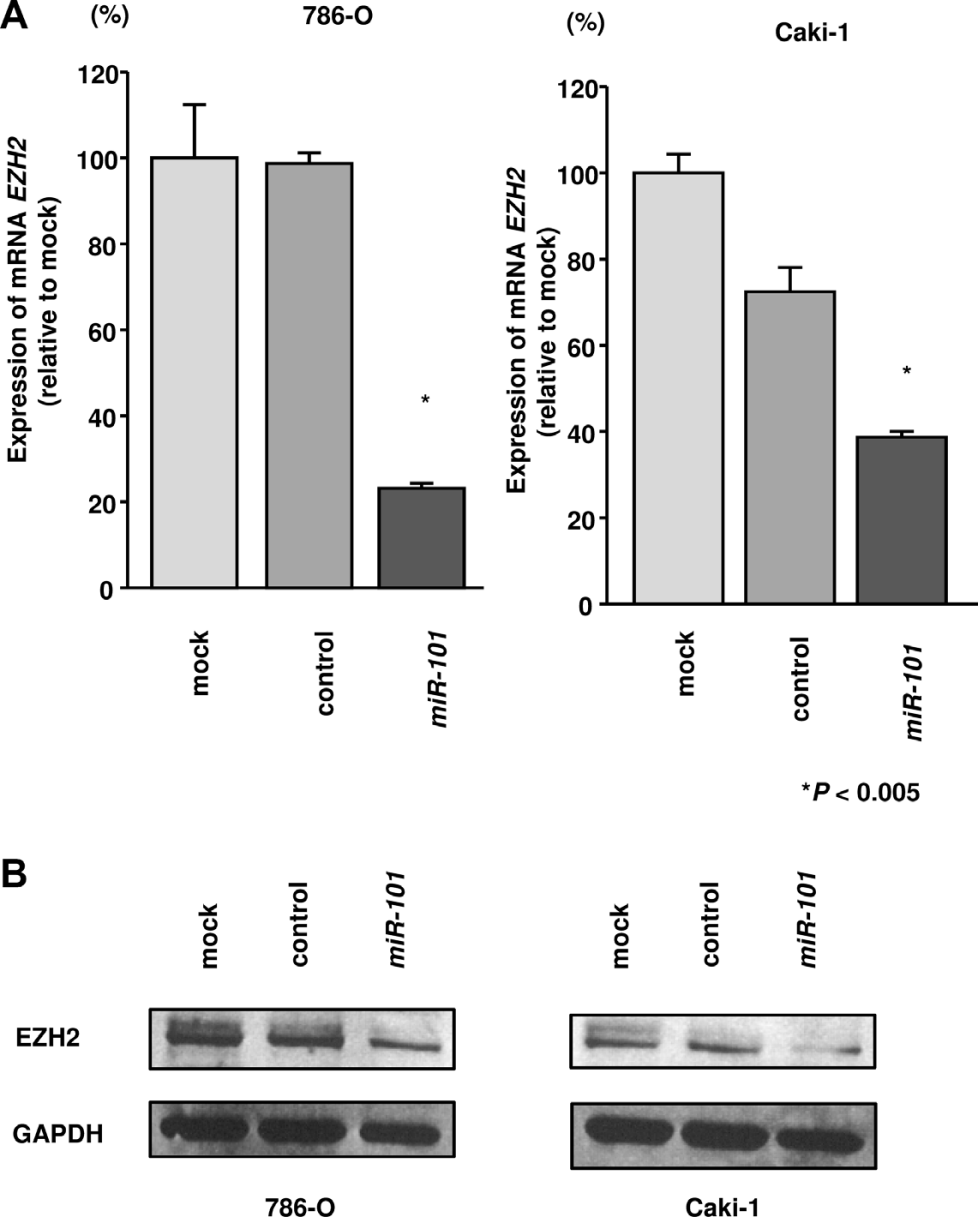
Effects of *miR-101* transfection on EZH2 mRNA and protein expression in RCC cells (**A**) *EZH2* mRNA expression was determined at 72 h after transfection with *miR-101*. *GUSB* was used as an internal control. (**B**) EZH2 protein expression was evaluated by western blotting at 72 h after transfection with *miR-101*. GAPDH was used as a loading control. *P < 0.005. The bars indicate SDs.

Next, we performed real-time RT-qPCR in 786- O and Caki-1 cells to analyze whether restoration of *miR- 101* altered the expression levels of the *UHRF1* and *EZH2* genes. Additionally, western blotting was carried out to investigate the effects of *miR-101* transfection on UHRF1 and EZH2 protein. The mRNA and protein expression levels of UHRF1 and EZH2 were significantly downregulated by *miR-101* transfection as compared with those in mock- or miR-control-transfected cells (*P* < 0.002 and *P* < 0.005; Figure 3A and 3B, Figure 4A and 4B). Because direct regulation of *EZH2* by *miR-101* in RCC has been reported by several groups [14, 15], we focused on the *UHRF1* gene in this study.

### miR-101 directly suppressed UHRF1 in RCC cells

We performed luciferase reporter assays in 786- O cells to determine whether *UHRF1* was directly suppressed by *miR-101*. The TargetScan database predicted that the putative *miR-101* target site in *UHRF1* was position 1030–1036 in the 3′UTR. We used two vectors: a vector encoding a partial wild-type sequence of the 3′ UTR of *UHRF1* mRNA including the predicted *miR-101* target site, and a vector lacking the *miR-101* target site. We found that the luminescence intensity was significantly reduced by cotransfection with *miR-101* and the vector carrying the wild-type 3′UTR of *UHRF1*. However, the luminescence intensity was not decreased when the seed sequence of the target site was deleted from the vectors (*P* < 0.0001; Figure 3C).

### Knockdown of UHRF1 significantly inhibited cell proliferation, migration, and invasion in RCC cell lines

**Figure 5:**
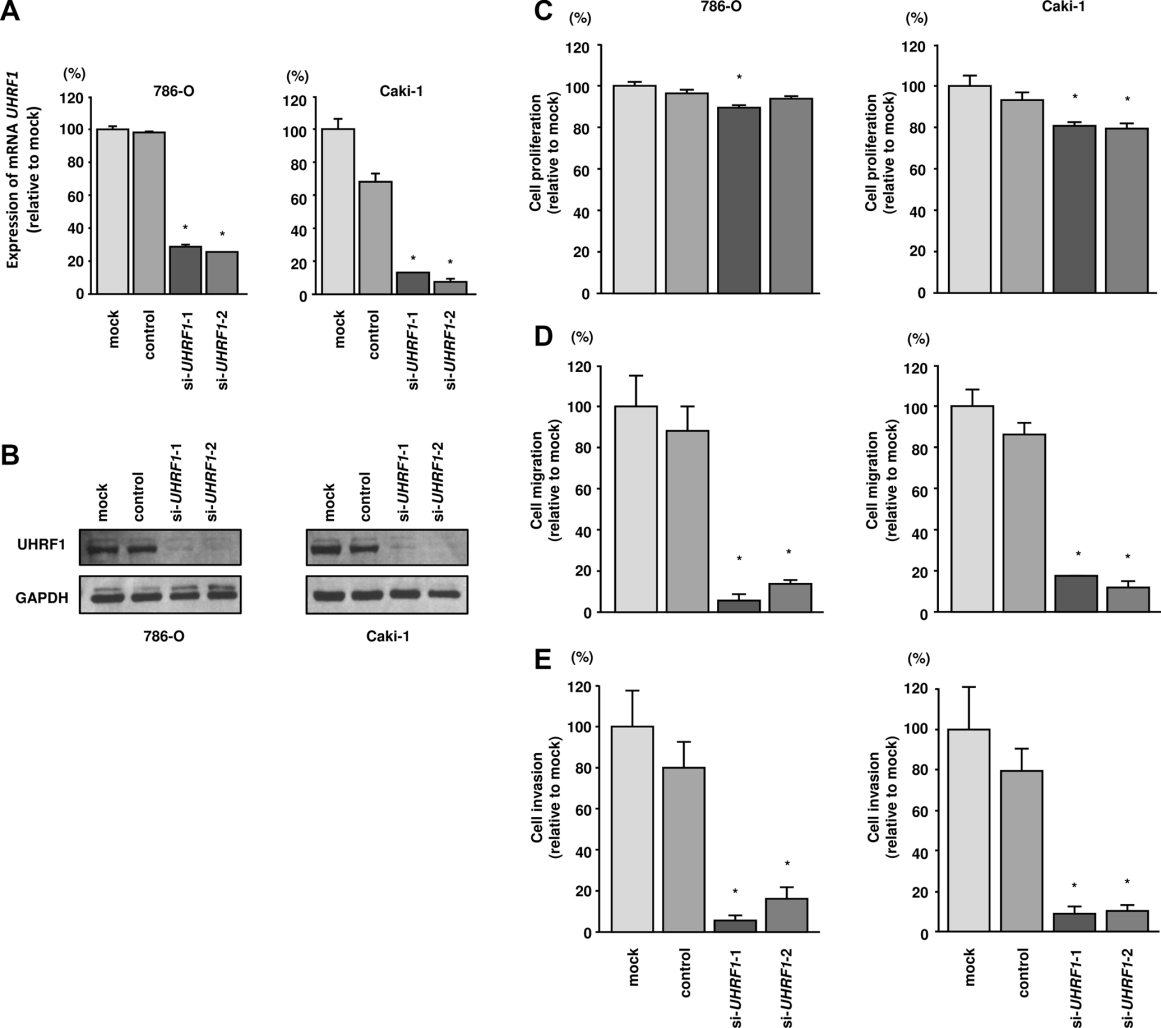
Effects of *UHRF1* knockdown in RCC cells and impact of UHRF1 expression on clinical RCC specimens (**A**) *UHRF1* mRNA expression was determined at 72 h after transfection with *si-UHRF1*. *GUSB* was used as an internal control. (**B**) UHRF1 protein expression was evaluated by western blotting at 72 h after transfection with *si-UHRF1*. GAPDH was used as a loading control. *P < 0.0001. The bars indicate SDs. (**C**) Cell proliferation was assessed 72 h after transfection with *si-UHRF1* using XTT assays. (**D**) Cell migration was assessed 48 h after transfection with *si-UHRF1* using uncoated Transwell polycarbonate membrane filters. (**E**) Cell invasion was assessed 48 h after transfection with *si-UHRF1* using Matrigel invasion assays. *P < 0.0001. The bars indicate SDs.

To investigate the functional role of *UHRF1* in RCC, we carried out loss-of-function studies by siRNA transfection. First, we evaluated the knockdown efficiency of si-*UHRF1* transfection in 786-O and Caki- 1 cells. RT-qPCR and western blotting indicated that si- *UHRF1* transfection effectively downregulated *UHRF1* and UHRF1 in 786-O and Caki-1 cells (*P* < 0.0001; Figure 5A and 5B).

In functional assays, si-*UHRF1* transfection significantly inhibited cell proliferation compared with that in mock- or si-control-transfected 786-O and Caki- 1 cells. Furthermore, cell migration and invasion were significantly inhibited by *si-UHRF1* transfection compared with mock- or si-control-transfection in 786-O and Caki-1 cells (*P* < 0.0001; Figure 5C–5E).

### Identification of pathways suppressed by UHRF1 knockdown in RCC cells

To further investigate which genes and pathways are suppressed by *miR-101/UHRF1* signaling, we performed genome wide gene expression analysis using knockdown of *UHRF1* by siRNA in 786-O cells. We deposited these data in the GEO (accession number: GSE77790). Genes which were significantly downregulated by *si-UHRF1* (Log2 [si-UHRF1/mock] < –1.0) were categorized by KEGG pathway analysis using the GeneCodis program. Table 5 shows pathways that were significantly downregulated by knockdown of *UHRF1*. Pathways related to post-transcriptional modification, including the nucleotide excision repair and mismatch repair pathways, were significantly suppressed by knockdown of *UHRF1*.

**Table 5:** Significantly downregulated pathways by knockdown of *UHRF1* in 786-O cells

KEGG number	Pathways	*p* value	Genes
4110	Cell cycle	1.20.E-14	*SMC1A, PTTG2, MCM4, CDC20, CCNA2, PTTG1, MCM7, CDC25C, CCNB1, MAD2L1, PRKDC, SKP1, MCM3, DBF4, STAG2, BUB1B, CDC45, MCM2, CDK1*
3030	DNA replication	3.77.E-11	*MCM4, MCM7, RFC1, POLD1, RFC5, MCM3, POLA2, FEN1, MCM2, RFC4*
4114	Oocyte meiosis	4.72.E-07	*CALML3, SMC1A, PTTG2, CDC20, PTTG1, CDC25C, CCNB1, MAD2L1, SKP1, CALM3, CDK1*
3018	RNA degradation	4.12.E-06	*DCPS, SKIV2L2, EXOSC3, PABPC1, PABPC3, LSM5, MPHOSPH6, DDX6*
3430	Mismatch repair	1.48.E-05	*RFC1, POLD1, EXO1, RFC5, RFC4*
5322	Systemic lupus erythematosus	3.52.E-05	*HIST1H2AI, SSB, H2AFX, HIST1H2AJ, HIST1H2AC, HIST1H4D, HIST1H3G, SNRPD1*
3013	RNA transport	4.26.E-05	*EIF5B, PABPC1, EIF3D, PABPC3, TACC3, EIF1AY, EIF3A, NUP205, EIF2S2, EIF1AX*
3420	Nucleotide excision repair	2.68.E-04	*CUL4B, RFC1, POLD1, RFC5, RFC4*
5130	Pathogenic *Escherichia coli* infection	9.75.E-04	*CLDN1, NCK2, TUBA1B, ITGB1, NCL*
533	Glycosaminoglycan biosynthesis - keratan sulfate	1.12.E-03	*CHST1, FUT8, B4GALT1*
4914	Progesterone-mediated oocyte maturation	1.38.E-03	*CCNA2, CDC25C, CCNB1, MAD2L1, HSP90AA1, CDK1*
4120	Ubiquitin mediated proteolysis	3.11.E-03	*CDC20, CUL4B, UBE3B, UBE3A, BRCA1, TRIP12, SKP1*

### UHRF1 and EZH2 expression in sunitinib-treated clinical RCC specimens

Finally, we examined UHRF1 expression status in clinical RCC specimens.

**Figure 6:**
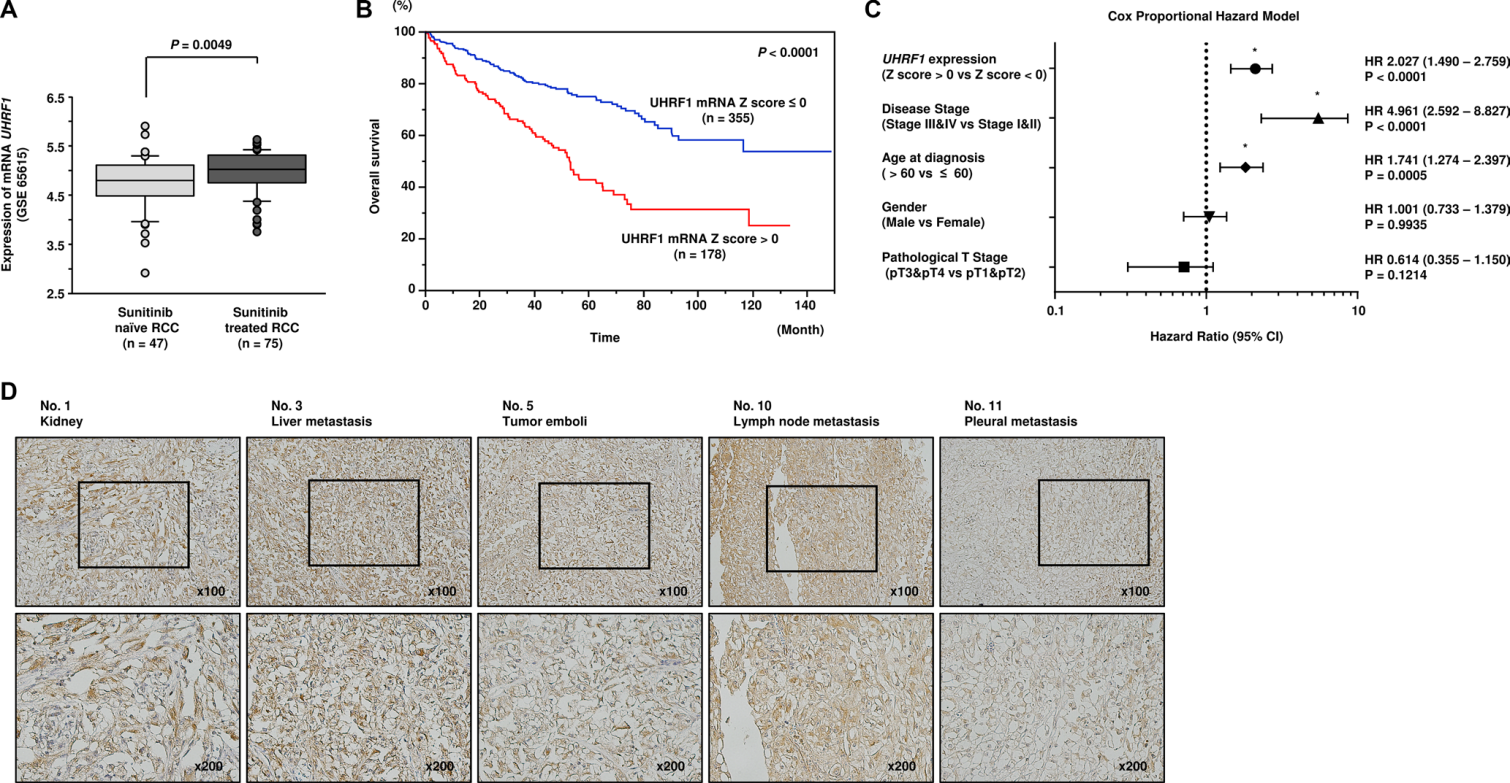
Clinical significance of *UHRF1* expression in RCC (**A**) *UHRF1* was highly expressed in sunitinib-treated RCC compared with that in sunitinib-naïve RCC (*P* = 0.0049). (**B**) The overall survival rate of patients with high *UHRF1* expression was significantly lower than that of patients with low *UHRF1* expression (*P* < 0.0001). (**C**) Multivariate Cox proportional hazards model for prediction of overall survival showed high *UHRF1* expression, advanced disease stage, and age at diagnosis were significant prognostic factors (*P* < 0.0001, *P* < 0.0001, *P* = 0.0005, respectively). (**D**) High expression of UHRF1 was observed in sunitinib-treated RCC specimens.

In a study with a relatively large sample size in GSE65615, higher *UHRF1* expression was observed in sunitinib-treated RCC specimens compared with that in sunitinib-naïve RCC specimens (*P* = 0.0049; Figure 6A). To examine whether *UHRF1* expression predicted overall survival, we used the TCGA-KIRC database (https://tcga-data.nci.nih.gov/tcga/). A total of 533 patients who underwent surgery for RCC and were pathologically diagnosed as having clear cell RCC were divided into two groups: z-score > 0 and z-score < 0 [16, 17]. Higher expression of *UHRF1* was associated with shorter overall survival (*P* < 0.0001; Figure 6B). Multivariate Cox proportional hazards models were used to assess independent predictors of overall survival times, including disease stage, pT stage, age at diagnosis, gender, and *UHRF1* expression. High *UHRF1* expression was one of the significant prognostic factors in patients with RCC (hazard ratio = 2.027, 95% confidence interval = 1.490– 2.759, *P* < 0.0001; Figure 6C). To analyze UHRF1 protein expression, immunohistochemistry was performed with sunitinib-treated specimens. Immunohistochemical staining of UHRF1 in these specimens demonstrated high expression of UHRF1 in sunitinib-treated RCC cells (Figure 6D).

**Figure 7:**
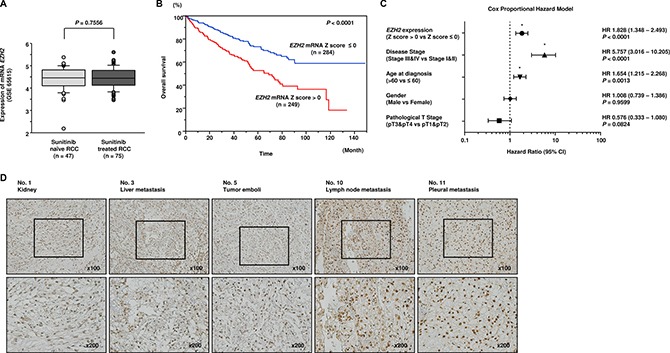
Clinical significance of *EZH2* expression in RCC (**A**) There was no significant difference of *EZH2* expression between sunitinib-treated RCC specimens and sunitinib-naïve RCC specimens. (**B**) The overall survival rate of patients with high *EZH2* expression was significantly lower than that of patients with low *EZH2* expression (*P* < 0.0001). (**C**) Multivariate Cox proportional hazards model for prediction of overall survival showed high *EZH2* expression, advanced disease stage, and age at diagnosis were significant prognostic factors (*P* < 0.0001, *P* < 0.0001, *P* = 0.0013, respectively). (**D**) High expression of EZH2 was observed in several sunitinib-treated RCC specimens.

Similarly, we analyzed *EZH2* status in clinical specimens. In GSE65615, we did not find significant difference of *EZH2* expression between sunitinib-treated RCC specimens and sunitinib-naïve RCC specimens (Figure 7A). Higher expression of *EZH2* was associated with shorter overall survival (*P* < 0.0001; Figure 7B). High *EZH2* expression was one of the significant prognostic factors in patients with RCC (hazard ratio = 1.828, 95% confidence interval = 1.348–2.493, *P* < 0.0001; Figure 7C). High expression of EZH2 was observed in several sunitinib-treated RCC specimens (Figure 7D).

## DISCUSSION

In patients treated with sunitinib, the initial response rate is approximately 40%; however, sunitinib-treated RCC cells usually acquire resistance to tyrosine kinase inhibitors [18]. Thus, overcoming sunitinib resistance is a major challenge for medical oncologists and urologists. Some mechanisms for sunitinib resistance in RCC have been described. One of the most well-studied pathways is hypoxia. VEGF-targeted drugs (e.g., sunitinib, pazopanib, axitinib, etc.) promote hypoxia in tumors by inhibiting angiogenesis, leading to high expression of HIF proteins [19]. Consequently, HIF proteins bind to hypoxia-responsive elements (HRE) and promote the expression of multiple oncogenes [20, 21]. Another mechanism is the activation of alternative signaling pathways. Hepatocyte growth factor (HGF)/cMET signaling, sphingosine kinase 1 (SPHK1)/sphingosine-1-phosphate (S1P)/extracellular signal-regulated kinase (ERK) signaling, and delta-like ligand4 (DLL4)/Notch signaling have significant roles in drug resistance [22–24].

Our research group has sequentially identified novel RCC oncogenic pathways based on antitumor miRNAs identified using RCC miRNA signatures [11, 13, 25, 26]. Our miRNA-mediated RNA network analysis may provide many insights into RCC pathogenesis. In this study, we constructed a miRNA expression signature using autopsy specimens from patients with RCC who showed sunitinib failure. We believe that this miRNA signature will contribute to analysis of the mechanisms mediating resistance to sunitinib treatment. We identified 40 microRNAs that were downregulated in sunitinib-treated RCC tissues compared with that in untreated RCC tissues. Among these downregulated miRNAs, we identified *miR-29b*, which has been reported to suppress the extracellular matrix (ECM). In RCC cells, *miR-29b* directly suppresses the lysyl oxidase-like 2 (LOXL2) gene, leading to inhibition of cancer cell invasion [25]. Furthermore, *miR-23b*, *miR- 10b*,*miR-135a*, *miR-29c*, *miR-27b*, *miR-1*, and *miR- 26b* have been reported as tumour-suppressive miRNAs in RCC by different research groups [12, 26–30]. In particular, *let-7c* sensitizes cells to 5-FU in RCC cells *in vitro*, and *miR-27b* sensitizes cells to doxorubicin, sorafenib, gefitinib in RCC cells [31, 32]. Chemoresistance and miRNAs listed in this signature have been reported previously in a variety of cancers: *miR-29b* in ovarian cancer, *miR-128* in breast cancer, *miR-23b* in gastric cancer, and *miR-10b* in colorectal and breast cancer [33–36]. Therefore, miRNAs listed in this signature would have importance in resistance to both chemotherapy and molecular targeted therapy. These facts ensure the reliability of the data in this signature.

In this study, we focused on the *miR-101* because *miR-101* was the most strongly downregulated miRNA in sunitinib-treated tissues. Our present data showed that *miR-101* functioned as an antitumor miRNA in RCC cells. The antitumor roles of *miR-101* have been reported in various types of cancers, including hepatocellular carcinoma, gastric cancer, breast cancer, and lung cancer [37–40]. Furthermore, *miR-101* restoration enhances chemosensitivity in lung cancer and salivary gland adenoid cystic carcinoma [41, 42]. Thus, our data are consistent with previous studies of *miR-101* in cancer research.

One interesting capacity of miRNA analyses is identification of miRNA-regulated genes in the human genome and investigation of the functional roles of these miRNA-regulated genes in cancer cells. In this study, we found that *UHRF1*, the master regulator of epigenetic modifications, was directly suppressed by *miR-101* in RCC cells and that its expression enhanced cancer cell migration and invasion. Moreover, overexpression of UHRF1 was confirmed in sunitinib-treated RCC tissues, and higher expression of *UHRF1* was associated with shorter overall survival after surgery for RCC. Because sunitinib is the first-line treatment option for recurrent RCC after surgical treatment, this association is consistent with other results. Ablation of *UHRF1* induces genomic hypomethylation, and overexpression of *UHRF1* has been reported in several cancers [43, 44]. UHRF1 consists of five recognizable domains: PHD, Tudor, SRA, RING, and UBL [45]. UHRF1 is required for DNA methyltransferase 1 (DNMT1) function through direct binding to DNMT1 and activation of DNMT1 function for maintenance of DNA methylation [46].

We found that *miR-101* directly suppressed *UHRF1*; however, previous reports have indicated that *EZH2* is also directly suppressed by *miR-101* [14, 47]. Consistent with this, we confirmed that EZH2 was suppressed by *miR-101* in RCC cells (Figure 4). Interestingly, EZH2 has also been reported to be a master regulator of transcription by modulation of histone modification or methylation [48, 49]. *miR-101* can target and suppress both *UHRF1* and *EZH2* in RCC cells. Previous report indicated that *UHRF1* and *EZH2* synergistically and independently silence tumor suppressors by methylation: *UHRF1* induce methylation of tumor suppressor gene DNA CpGs and H3-K9me3, and *EZH2* induce methylation of H3-K27 [50]. Overexpression of both *UHRF1* and *EZH2* coordinately suppressed antitumor genes and contributed to prostate cancer pathogenesis and metastasis [50]. More recently, patient-derived clear cell RCC xenografts with sunitinib resistant phenotype showed increased EZH2 expression, and inhibition of *EZH2* resulted in enhancement of the antitumor effects of sunitinib [51]. Consequently, we speculate that overexpression of *UHRF1* and *EZH2* coordinately suppressed antitumor genes and deeply contribute to sunitinib resistant processes in RCC cells. In sunitinib-treated RCC tissues, loss of antitumor *miR- 101* may lead to upregulation of *UHRF1* and *EZH2*, and consequently, post-transcriptional modification of multiple genes would promote cancer-related phenotypes in cells. Moreover, we investigated the downstream pathways suppressed by knockdown of *UHRF1* and found that pathways such as cell cycle, DNA replication, and RNA degradation were enriched in RCC cells. Thus, antitumor *miR-101*-mediated *UHRF1* pathways may be suppressed by sunitinib treatment. Our present data will contribute to our understanding of drug-resistance mechanisms in RCC cells.

## MATERIALS AND METHODS

### Patients and clinical RCC specimens

Clinical kidney specimens were obtained from patients admitted to Kagoshima University Hospital and Teikyo University Chiba Medical Centre Hospital from 2004 to 2014. A total of 42 pairs of clear cell renal carcinoma and adjacent noncancerous tissue were obtained by nephrectomy. Three patients who died of RCC after sunitinib treatment failure underwent autopsies. RCC tissues (*n* = 42), adjacent noncancerous kidney tissues (*n* = 41), and sunitinib-treated RCC tissues (*n*= 11) were used. The patients' backgrounds and characteristics are summarized in Tables 1 and 3. Samples were staged according to the UICC TNM classification. Written consent for tissue donation for research purposes was obtained from each patient before sample collection. The protocol was approved by the Institutional Review Board of Chiba University, Kagoshima University and Teikyo University.

### Construction of the miRNA expression signature of sunitinib-treated RCC

miRNA expression patterns were evaluated using a TaqMan LDA Human microRNA Panel v2.0 (Applied Biosystems, Foster City, CA, USA). A cut-off *P*-value of less than 0.05 was used to narrow down the candidates after global normalization of the raw data. After global normalization, additional normalization was carried out with the *U6* gene. The procedure was performed as described previously [11, 52].

### Cell culture

Human RCC cell lines (786-O and Caki-1 cells) were obtained from the American Type Culture Collection (Manassas, VA, USA). Cells were maintained in RPMI-1640 medium supplemented with 10% fetal bovine serum in a humidified atmosphere containing 5% CO_2_ and 95% air at 37°C.

### RNA isolation

Total RNA was isolated using TRIzol reagent (Invitrogen, Carlsbad, CA, USA). The quality of RNA was confirmed using an Agilent 2100 Bioanalyzer (Agilent Technologies) as described previously [52–54].

### Quantitative real-time reverse transcription polymerase chain reaction (RT-qPCR)

The expression levels of *miR-101* (Assay ID: 002253) were analyzed by TaqMan RT-qPCR (TaqMan MicroRNA Assay; Applied Biosystems) and normalized to *RNU48* expression (Assay ID: 001006). TaqMan probes and primers for *UHRF1*(P/N: Hs01086727_m1), *EZH2* (P/N: Hs01016789_m1), and *GAPDH* (P/N: Hs02758991_g1) as an internal control were obtained from Applied Biosystems (Assay-On-Demand Gene Expression Products). The procedure was carried out as previously described [52, 53].

### Transfection with miRNA mimic and small-interfering RNA (siRNA)

Ambion Pre-miR miRNA precursor for *hsa-miR-101-3p* (product ID: PM11414) was used in this study as a miRNA mimic. The following siRNAs were used: Stealth Select RNAi siRNA; si-*UHRF1* (cat no.: HSS179005 and HSS179006; Invitrogen); and negative control miRNA/siRNA (P/N: AM17110; Applied Biosystems). RNAs were incubated with OPTI-MEM (Invitrogen) and Lipofectamine RNAiMAX reagent (Invitrogen). The transfection procedures were performed as previously described [52, 53].

### Cell proliferation, migration, and invasion assays

Cell proliferation assays were performed using XTT assays, migration assays were performed using uncoated Transwell polycarbonate membrane filters, and invasion assays were performed using Matrigel-coated Boyden chambers, as previously described [52, 53].

### Identification of genes suppressed by miR-101

A combination of *in silico* and genome-wide gene expression analyses were carried out to investigate target genes suppressed by miR-101. First, genes suppressed by *miR-101* were listed using the TargetScan database. Next, to identify upregulated genes in RCC, we analyzed a publicly available gene expression data set in the Gene Expression Omnibus (GEO; accession numbers: GSE36985 and GSE22541). Finally, upregulated mRNAs containing *miR- 101* target sites were listed as putative target genes of *miR-101*. The procedure for selection is summarized in Figure 2.

### Western blotting

Immunoblotting was performed with rabbit anti-UHRF1 antibodies (1:1000, PA5-29884; Pierce Antibodies, Thermo Scientific, Fremont, CA, USA) and anti-EZH2 antibodies (1:250, 36-6300; Life Technologies, Carlsbad, CA, USA). Anti-GAPDH antibodies (1:1000, ab8245; Abcam, Cambridge, UK) were used as an internal loading control. Membranes were washed and incubated with anti-rabbit IgG horseradish peroxidase (HRP)-linked antibodies (7074; Cell Signaling Technology, Danvers, MA, USA). Complexes were visualized with Clarity Western ECL Substrate (Bio-Rad, Hercules, CA, USA). The procedures were performed as previously described [52, 53].

### Plasmid construction and dual-luciferase reporter assay

Partial wild-type sequences of the *UHRF1* 3′ untranslated region (UTR) or those with a deleted *miR-101* target site (position 1030–1036 of the *UHRF1* 3′ UTR) were inserted between the *Xho*I–*Pme*I restriction sites in the 3′ UTR of the *hRluc* gene in the psiCHECK-2 vector (C8021; Promega, Madison, WI, USA). The protocol for vector construction was described previously [52, 53].

### Identification of pathways and genes suppressed by UHRF1 in RCC

To identify molecular pathways suppressed by *UHRF1* gene expression in RCC cells, we performed gene expression analysis using si-*UHRF1*-transfected 786-O cells. An oligomicroarray (SurePrint G3 Human 8×60k v3; Agilent Technologies) was used for gene expression studies. The data were deposited in the GEO database (accession number GSE77790). Genes downregulated by knockdown of UHRF1 were categorized into Kyoto Encyclopaedia of Genes and Genomes (KEGG) pathways using the GENECODIS program (http://genecodis.cnb.csic.es/). The strategy of this analysis procedure has been described previously [52–54].

### Immunohistochemisty

A total of 11 specimens were used (Table 1). Tissue specimens were immunostained with an Ultra-Vision Detection System (Thermo Scientific) following the manufacturer's protocol. Primary rabbit polyclonal antibodies against UHRF1 (1:500, PA5-29884; Pierce Antibodies, Thermo Scientific) and EZH2 (1:125, 36-6300; Life Technologies) were used for immunochemistry. The slides were treated with biotinylated goat antibodies (Histofine SAB-PO kit; Nichirei, Tokyo, Japan). The procedures were performed as previously described [52–54].

### TCGA-KIRC and other human RCC data analysis

To explore the clinical significance of *UHRF1* in RCC, we used the RNA sequencing database in TCGA-KIRC (The Cancer Genome Atlas Kidney Renal Clear Cell Carcinoma: https://tcga-data.nci.nih.gov/tcga/). The gene expression and clinical data were retrieved from cBioportal (http://www.cbioportal.org/, the provisional data downloaded on May 10th, 2016). The normalized mRNA expression value in the RNA sequencing data was processed and provided in Z-score. We performed multivariate analysis (Cox proportional hazards model) that included pathological tumour and disease stage, age, and gender under consideration. We also employed the gene expression microarray data including sunitinib-treated and sunitinib-naïve human RCC specimens (GSE 65615).

### Statistical analysis

The relationships between 2 groups and numerical values were analyzed using Mann-Whitney *U*-tests. The relationships among more than 3 variables and numerical values were analyzed using the Bonferroni-adjusted Mann-Whitney *U*-test. A multivariate Cox proportional hazards model was used to establish independent factors for overall survival. Survival analysis was carried out using the Kaplan–Meier method and log-rank tests. The Kaplan–Meier method and log-rank test were performed using JMP software (version 12, SAS Institute Inc., Cary, NC, USA); all other analyses were performed using Expert StatView (version 5, SAS Institute Inc.).

## SUPPLEMENTARY MATERIALS TABLE



## References

[1] Ferlay J, Soerjomataram I, Dikshit R, Eser S, Mathers C, Rebelo M, Parkin DM, Forman D, Bray F (2015). Cancer incidence and mortality worldwide: sources, methods and major patterns in GLOBOCAN 2012. Int J Cancer.

[2] Motzer RJ, Jonasch E, Agarwal N, Beard C, Bhayani S, Bolger GB, Chang SS, Choueiri TK, Costello BA, Derweesh IH, Gupta S, Hancock SL, Kim JJ (2015). Kidney cancer, version 3. 2015. J Natl Compr Canc Netw.

[3] Motzer RJ, Hutson TE, Tomczak P, Michaelson MD, Bukowski RM, Rixe O, Oudard S, Negrier S, Szczylik C, Kim ST, Chen I, Bycott PW, Baum CM (2007). Sunitinib versus interferon alfa in metastatic renal-cell carcinoma. N Engl J Med.

[4] Tomita Y (2016). Treatment strategies for advanced renal cell carcinoma: A new paradigm for surgical treatment. Int J Urol.

[5] Porta C, Paglino C, Grunwald V (2014). Sunitinib re-challenge in advanced renal-cell carcinoma. Br J Cancer.

[6] Eisen T, Sternberg CN, Robert C, Mulders P, Pyle L, Zbinden S, Izzedine H, Escudier B (2012). Targeted therapies for renal cell carcinoma: review of adverse event management strategies. J Natl Cancer Inst.

[7] Goto Y, Kurozumi A, Enokida H, Ichikawa T, Seki N (2015). Functional significance of aberrantly expressed microRNAs in prostate cancer. Int J Urol.

[8] Bartel DP (2009). MicroRNAs: target recognition and regulatory functions. Cell.

[9] Garzon R, Calin GA, Croce CM (2009). MicroRNAs in Cancer. Annu Rev Med.

[10] Tay Y, Rinn J, Pandolfi PP (2014). The multilayered complexity of ceRNA crosstalk and competition. Nature.

[11] Hidaka H, Seki N, Yoshino H, Yamasaki T, Yamada Y, Nohata N, Fuse M, Nakagawa M, Enokida H (2012). Tumor suppressive microRNA-1285 regulates novel molecular targets: aberrant expression and functional significance in renal cell carcinoma. Oncotarget.

[12] Ishihara T, Seki N, Inoguchi S, Yoshino H, Tatarano S, Yamada Y, Itesako T, Goto Y, Nishikawa R, Nakagawa M, Enokida H (2014). Expression of the tumor suppressive miRNA-23b/27b cluster is a good prognostic marker in clear cell renal cell carcinoma. J Urol.

[13] Yoshino H, Enokida H, Itesako T, Kojima S, Kinoshita T, Tatarano S, Chiyomaru T, Nakagawa M, Seki N (2013). Tumor-suppressive microRNA-143/145 cluster targets hexokinase-2 in renal cell carcinoma. Cancer Sci.

[14] Sakurai T, Bilim VN, Ugolkov AV, Yuuki K, Tsukigi M, Motoyama T, Tomita Y (2012). The enhancer of zeste homolog 2 (EZH2), a potential therapeutic target, is regulated by miR-101 in renal cancer cells. Biochem Biophys Res Commun.

[15] Zheng M, Jiang YP, Chen W, Li KD, Liu X, Gao SY, Feng H, Wang SS, Jiang J, Ma XR, Cen X, Tang YJ, Chen Y (2015). Snail and Slug collaborate on EMT and tumor metastasis through miR-101-mediated EZH2 axis in oral tongue squamous cell carcinoma. Oncotarget.

[16] Gao J, Aksoy BA, Dogrusoz U, Dresdner G, Gross B, Sumer SO, Sun Y, Jacobsen A, Sinha R, Larsson E, Cerami E, Sander C, Schultz N (2013). Integrative analysis of complex cancer genomics and clinical profiles using the cBioPortal. Sci Signal.

[17] Cerami E, Gao J, Dogrusoz U, Gross BE, Sumer SO, Aksoy BA, Jacobsen A, Byrne CJ, Heuer ML, Larsson E, Antipin Y, Reva B, Goldberg AP (2012). The cBio cancer genomics portal: an open platform for exploring multidimensional cancer genomics data. Cancer Discov.

[18] Powles T, Staehler M, Ljungberg B, Bensalah K, Canfield SE, Dabestani S, Giles R, Hofmann F, Hora M, Kuczyk MA, Lam T, Marconi L, Merseburger AS (2016). Updated EAU Guidelines for Clear Cell Renal Cancer Patients Who Fail VEGF Targeted Therapy. Eur Urol.

[19] Joosten SC, Hamming L, Soetekouw PM, Aarts MJ, Veeck J, van Engeland M, Tjan-Heijnen VC (2015). Resistance to sunitinib in renal cell carcinoma: From molecular mechanisms to predictive markers and future perspectives. Biochim Biophys Acta.

[20] Harris AL (2002). Hypoxia—a key regulatory factor in tumour growth. Nat Rev Cancer.

[21] Baldewijns MM, van Vlodrop IJ, Vermeulen PB, Soetekouw PM, van Engeland M, de Bruine AP (2010). VHL and HIF signalling in renal cell carcinogenesis. J Pathol.

[22] Shojaei F, Lee JH, Simmons BH, Wong A, Esparza CO, Plumlee PA, Feng J, Stewart AE, Hu-Lowe DD, Christensen JG (2010). HGF/c-Met acts as an alternative angiogenic pathway in sunitinib-resistant tumors. Cancer Res.

[23] Zhang L, Wang X, Bullock AJ, Callea M, Shah H, Song J, Moreno K, Visentin B, Deutschman D, Alsop DC, Atkins MB, Mier JW, Signoretti S (2015). Anti-S1P Antibody as a Novel Therapeutic Strategy for VEGFR TKI-Resistant Renal Cancer. Clin Cancer Res.

[24] Li JL, Sainson RC, Oon CE, Turley H, Leek R, Sheldon H, Bridges E, Shi W, Snell C, Bowden ET, Wu H, Chowdhury PS, Russell AJ (2011). DLL4-Notch signaling mediates tumor resistance to anti-VEGF therapy *in vivo*. Cancer Res.

[25] Nishikawa R, Chiyomaru T, Enokida H, Inoguchi S, Ishihara T, Matsushita R, Goto Y, Fukumoto I, Nakagawa M, Seki N (2015). Tumour-suppressive microRNA-29s directly regulate LOXL2 expression and inhibit cancer cell migration and invasion in renal cell carcinoma. FEBS Lett.

[26] Kurozumi A, Kato M, Goto Y, Matsushita R, Nishikawa R, Okato A, Fukumoto I, Ichikawa T, Seki N (2016). Regulation of the collagen cross-linking enzymes LOXL2 and PLOD2 by tumor-suppressive microRNA-26a/b in renal cell carcinoma. Int J Oncol.

[27] Liu W, Zabirnyk O, Wang H, Shiao YH, Nickerson ML, Khalil S, Anderson LM, Perantoni AO, Phang JM (2010). miR-23b targets proline oxidase, a novel tumor suppressor protein in renal cancer. Oncogene.

[28] Fritz HK, Lindgren D, Ljungberg B, Axelson H, Dahlback B (2014). The miR(21/10b) ratio as a prognostic marker in clear cell renal cell carcinoma. Eur J Cancer.

[29] Kawakami K, Enokida H, Chiyomaru T, Tatarano S, Yoshino H, Kagara I, Gotanda T, Tachiwada T, Nishiyama K, Nohata N, Seki N, Nakagawa M (2012). The functional significance of miR-1 and miR-133a in renal cell carcinoma. Eur J Cancer.

[30] Yamada Y, Hidaka H, Seki N, Yoshino H, Yamasaki T, Itesako T, Nakagawa M, Enokida H (2013). Tumor-suppressive microRNA-135a inhibits cancer cell proliferation by targeting the c-MYC oncogene in renal cell carcinoma. Cancer Sci.

[31] Mu W, Hu C, Zhang H, Qu Z, Cen J, Qiu Z, Li C, Ren H, Li Y, He X, Shi X, Hui L (2015). miR-27b synergizes with anticancer drugs via p53 activation and CYP1B1 suppression. Cell Res.

[32] Peng J, Mo R, Ma J, Fan J (2015). let-7b and let-7c are determinants of intrinsic chemoresistance in renal cell carcinoma. World J Surg Oncol.

[33] Dai F, Zhang Y, Chen Y (2014). Involvement of miR-29b signaling in the sensitivity to chemotherapy in patients with ovarian carcinoma. Hum Pathol.

[34] Zhu Y, Yu F, Jiao Y, Feng J, Tang W, Yao H, Gong C, Chen J, Su F, Zhang Y, Song E (2011). Reduced miR-128 in breast tumor-initiating cells induces chemotherapeutic resistance via Bmi-1 and ABCC5. Clin Cancer Res.

[35] An Y, Zhang Z, Shang Y, Jiang X, Dong J, Yu P, Nie Y, Zhao Q (2015). miR-23b-3p regulates the chemoresistance of gastric cancer cells by targeting ATG12 and HMGB2. Cell Death Dis.

[36] Nishida N, Yamashita S, Mimori K, Sudo T, Tanaka F, Shibata K, Yamamoto H, Ishii H, Doki Y, Mori M (2012). MicroRNA-10b is a prognostic indicator in colorectal cancer and confers resistance to the chemotherapeutic agent 5-fluorouracil in colorectal cancer cells. Ann Surg Oncol.

[37] Su H, Yang JR, Xu T, Huang J, Xu L, Yuan Y, Zhuang SM (2009). MicroRNA-101, down-regulated in hepatocellular carcinoma, promotes apoptosis and suppresses tumorigenicity. Cancer Res.

[38] Wang HJ, Ruan HJ, He XJ, Ma YY, Jiang XT, Xia YJ, Ye ZY, Tao HQ (2010). MicroRNA-101 is down-regulated in gastric cancer and involved in cell migration and invasion. Eur J Cancer.

[39] Li JT, Jia LT, Liu NN, Zhu XS, Liu QQ, Wang XL, Yu F, Liu YL, Yang AG, Gao CF (2015). MiRNA-101 inhibits breast cancer growth and metastasis by targeting CX chemokine receptor 7. Oncotarget.

[40] Wang L, Zhang LF, Wu J, Xu SJ, Xu YY, Li D, Lou JT, Liu MF (2014). IL-1beta-mediated repression of microRNA-101 is crucial for inflammation-promoted lung tumorigenesis. Cancer Res.

[41] Riquelme E, Suraokar M, Behrens C, Lin HY, Girard L, Nilsson MB, Simon G, Wang J, Coombes KR, Lee JJ, Hong WK, Heymach J, Minna JD (2014). VEGF/VEGFR-2 upregulates EZH2 expression in lung adenocarcinoma cells and EZH2 depletion enhances the response to platinum-based and VEGFR-2-targeted therapy. Clin Cancer Res.

[42] Liu XY, Liu ZJ, He H, Zhang C, Wang YL (2015). MicroRNA-101-3p suppresses cell proliferation, invasion and enhances chemotherapeutic sensitivity in salivary gland adenoid cystic carcinoma by targeting Pim-1. Am J Cancer Res.

[43] Unoki M, Brunet J, Mousli M (2009). Drug discovery targeting epigenetic codes: the great potential of UHRF1, which links DNA methylation and histone modifications, as a drug target in cancers and toxoplasmosis. Biochem Pharmacol.

[44] Matsushita R, Yoshino H, Enokida H, Goto Y, Miyamoto K, Yonemori M, Inoguchi S, Nakagawa M, Seki N (2016). Regulation of UHRF1 by dual-strand tumor-suppressor microRNA-145 (miR-145-5p and miR-145-3p): Inhibition of bladder cancer cell aggressiveness. Oncotarget.

[45] Hu L, Li Z, Wang P, Lin Y, Xu Y (2011). Crystal structure of PHD domain of UHRF1 and insights into recognition of unmodified histone H3 arginine residue 2. Cell Res.

[46] Bashtrykov P, Jankevicius G, Jurkowska RZ, Ragozin S, Jeltsch A (2014). The UHRF1 protein stimulates the activity and specificity of the maintenance DNA methyltransferase DNMT1 by an allosteric mechanism. J Biol Chem.

[47] Varambally S, Cao Q, Mani RS, Shankar S, Wang X, Ateeq B, Laxman B, Cao X, Jing X, Ramnarayanan K, Brenner JC, Yu J, Kim JH (2008). Genomic loss of microRNA-101 leads to overexpression of histone methyltransferase EZH2 in cancer. Science.

[48] Kim KH, Roberts CW (2016). Targeting EZH2 in cancer. Nat Med.

[49] Chase A, Cross NC (2011). Aberrations of EZH2 in cancer. Clin Cancer Res.

[50] Babbio F, Pistore C, Curti L, Castiglioni I, Kunderfranco P, Brino L, Oudet P, Seiler R, Thalman GN, Roggero E, Sarti M, Pinton S, Mello-Grand M (2012). The SRA protein UHRF1 promotes epigenetic crosstalks and is involved in prostate cancer progression. Oncogene.

[51] Adelaiye R, Ciamporcero E, Miles KM, Sotomayor P, Bard J, Tsompana M, Conroy D, Shen L, Ramakrishnan S, Ku SY, Orillion A, Prey J, Fetterly G (2015). Sunitinib dose escalation overcomes transient resistance in clear cell renal cell carcinoma and is associated with epigenetic modifications. Mol Cancer Ther.

[52] Goto Y, Kojima S, Nishikawa R, Kurozumi A, Kato M, Enokida H, Matsushita R, Yamazaki K, Ishida Y, Nakagawa M, Naya Y, Ichikawa T, Seki N (2015). MicroRNA expression signature of castration-resistant prostate cancer: the microRNA-221/222 cluster functions as a tumour suppressor and disease progression marker. Br J Cancer.

[53] Goto Y, Kojima S, Kurozumi A, Kato M, Okato A, Matsushita R, Ichikawa T, Seki N (2016). Regulation of E3 ubiquitin ligase-1 (WWP1) by microRNA-452 inhibits cancer cell migration and invasion in prostate cancer. Br J Cancer.

[54] Kurozumi A, Goto Y, Matsushita R, Fukumoto I, Kato M, Nishikawa R, Sakamoto S, Enokida H, Nakagawa M, Ichikawa T, Seki N (2016). Tumor-suppressive microRNA-223 inhibits cancer cell migration and invasion by targeting ITGA3/ITGB1 signaling in prostate cancer. Cancer Sci.

