# Supplementary material for: The microRNA signature of patients with sunitinib failure: regulation of UHRF1 pathways by microRNA-101 in renal cell carcinoma
Source: Oncotarget. 2016 Jul 28;7(37):59070–86. doi: 10.18632/oncotarget.10887 (PMC5312296; doi:10.18632/oncotarget.10887)
Supplement: Supplementary file 1 [file oncotarget-07-59070-s001.pdf]

The microRNA signature of patients with sunitinib failure: regulation of *UHRF1* pathways by *microRNA-101* in renal cell carcinoma

Supplementary Materials

Supplementary Table S1: Downregulated miRNAs in primary RCC (versus normal kidney tissue)

miRNA	Log2 ratio (primary/normal)	Normal kidney tissue	Primary RCC	*P*-value
hsa-miR-184	–8.31	6.07.E-04	1.91.E-06	0.03807
hsa-miR-888	–7.61	1.97.E-04	1.00.E-06	0.02562
hsa-miR-508-3p	–6.43	6.65.E-04	7.73.E-06	0.00320
hsa-miR-1251	–5.46	2.42.E-05	5.48.E-07	0.02975
hsa-miR-200c	–5.37	2.32.E-02	5.61.E-04	0.00044
hsa-miR-509-5p	–5.26	3.97.E-04	1.03.E-05	0.03020
hsa-miR-141	–5.19	2.01.E-03	5.50.E-05	0.00050
*hsa-miR-30c-2**	–4.40	4.14.E-05	1.97.E-06	0.03614
hsa-miR-504	–3.97	4.28.E-05	2.74.E-06	0.00764
hsa-miR-133b	–3.75	1.47.E-04	1.10.E-05	0.01808
hsa-miR-135a	–3.69	7.69.E-03	5.96.E-04	0.01813
hsa-miR-206	–3.68	4.15.E-05	3.24.E-06	0.04714
hsa-miR-299-5p	–3.61	1.14.E-05	9.36.E-07	0.04543
hsa-miR-337-5p	–3.55	1.27.E-04	1.09.E-05	0.00351
hsa-miR-654-3p	–3.46	1.76.E-05	1.59.E-06	0.03895
*hsa-miR-10a**	–2.73	5.57.E-05	8.41.E-06	0.00422
hsa-miR-23b	–2.54	2.01.E-03	3.46.E-04	0.00066
hsa-miR-204	–2.39	1.76.E-01	3.38.E-02	0.03718
hsa-miR-1285	–2.34	2.53.E-04	5.01.E-05	0.03807
hsa-miR-218	–2.20	1.18.E-02	2.57.E-03	0.02862
*hsa-miR-136**	–2.03	1.94.E-04	4.75.E-05	0.01162
*hsa-miR-214**	–2.02	1.18.E-03	2.93.E-04	0.00262
hsa-miR-335	–1.98	1.30.E-03	3.30.E-04	0.00457
*hsa-miR-335**	–1.94	3.37.E-04	8.76.E-05	0.04749
hsa-miR-655	–1.94	7.64.E-05	1.99.E-05	0.00450
hsa-miR-429	–1.94	1.61.E-02	4.20.E-03	0.00499
hsa-miR-133a	–1.92	1.80.E-03	4.75.E-04	0.01219
hsa-miR-363	–1.82	4.14.E-04	1.17.E-04	0.01846
hsa-miR-200b	–1.79	5.82.E-02	1.68.E-02	0.00014
hsa-miR-149	–1.78	1.98.E-03	5.75.E-04	0.02698
hsa-miR-214	–1.74	9.60.E-03	2.87.E-03	0.01435
*hsa-miR-26a-1**	–1.72	1.07.E-04	3.25.E-05	0.00056
hsa-miR-10a	–1.64	1.42.E-02	4.58.E-03	0.00019
hsa-miR-127-3p	–1.63	1.62.E-03	5.22.E-04	0.01017
*hsa-miR-27b**	–1.63	1.33.E-04	4.28.E-05	0.02970
hsa-miR-199a-3p	–1.62	4.17.E-02	1.36.E-02	0.02286
*hsa-miR-10b**	–1.60	8.03.E-03	2.64.E-03	0.00050
hsa-miR-660	–1.54	2.68.E-02	9.21.E-03	0.00562
hsa-miR-376c	–1.51	8.66.E-04	3.05.E-04	0.02709
hsa-miR-532-3p	–1.50	7.42.E-03	2.63.E-03	0.01591
